# OLFM2 promotes epithelial-mesenchymal transition, migration, and invasion in colorectal cancer through the TGF-β/Smad signaling pathway

**DOI:** 10.1186/s12885-024-11925-3

**Published:** 2024-02-13

**Authors:** Yong Tang, Yi Liu, Xiaobo Wang, Haiyang Guo, Lin Chen, Guangbing Hu, Yutong Cui, Shiqi Liang, Ji Zuo, Zichen Luo, Xinrui Chen, Xianfei Wang

**Affiliations:** 1 Department of Gastroenterology, Affiliated Hospital of North Sichuan Medical College, Nanchong, China; 2 Digestive Endoscopy Center, Affiliated Hospital of North Sichuan Medical College, Nanchong, China; 3 Department of Radiation Oncology, Chongqing University Cancer Hospital, Chongqing, China; 4 Department of Gastroenterology, Ziyang Yanjiang People’s Hospital, Ziyang, China

**Keywords:** OLFM2, EMT, TGF-β/Smad signaling pathway, Colorectal cancer

## Abstract

**Background:**

Colorectal cancer (CRC) is an aggressive tumor of the gastrointestinal tract, which is a major public health concern worldwide. Despite numerous studies, the precise mechanism of metastasis behind its progression remains elusive. As a member of the containing olfactomedin domains protein family, olfactomedin 2 (OLFM2) may play a role in tumor metastasis. It is highly expressed in colorectal cancer, and its role in the metastasis of CRC is still unclear. As such, this study seeks to explore the function of OLFM2 on CRC metastasis and its potential mechanisms.

**Methods:**

Real-time fluorescence quantitative PCR and western blotting were used to study the expression of OLFM2 in human CRC and adjacent normal tissues. Knockdown and overexpression OLFM2 cell lines were constructed using siRNA and overexpression plasmids to explore the role of OLFM2 in the migration and invasion of CRC through transwell, and wound healing experiments. Finally, the expression of epithelial-mesenchymal transition (EMT) -related proteins and TGF-β/Smad signaling pathway-related proteins was investigated using western blotting.

**Results:**

In this study, we observed an elevation of OLFM2 expression levels in CRC tissues. To investigate the function of OLFM2, we overexpressed and knocked down OLFM2. We discovered that OLFM2 knockdown inhibited migration and invasion of colon cancer cells. Furthermore, E-cadherin expression increased while N-cadherin and Vimentin expression were opposite. It is no surprise that overexpressing OLFM2 had the opposite effects. We also identified that OLFM2 knockdown resulted in reduced TGF-βR1 and downstream molecules p-Smad2 and p-Smad3, which are related to the TGF-β / Smad pathway. In contrast, overexpressing OLFM2 significantly boosted their expression levels.

**Conclusion:**

The protein OLFM2 has been identified as a crucial determinant in the progression of CRC. Its mechanism of action involves the facilitation of EMT through the TGF-β/Smad signaling pathway. Given its pivotal role in CRC, OLFM2 has emerged as a promising diagnostic and therapeutic target for the disease. These results indicate the potential of OLFM2 as a valuable biomarker for CRC diagnosis and treatment and highlight the need for further research exploring its clinical significance.

**Supplementary Information:**

The online version contains supplementary material available at 10.1186/s12885-024-11925-3.

## Introduction

CRC is a significant global health issue, causing approximately 900,000 deaths annually and ranking fourth in terms of mortality rates. This problem is especially severe in developing countries [1]. The primary treatment for CRC is surgical resection, which has the potential of curing the disease. However, disease recurrence affects around 20% of surgical patients, leading to metastasis and ultimately, death [2–4]. As such, it is essential to investigate the underlying mechanisms of colorectal cancer metastasis to improve patient outcomes.

As tumors progress, cells undergo a crucial transformation known as Epithelial-Mesenchymal Transition (EMT), granting them the ability to migrate and invade, ultimately leading to malignancy [5–7]. Extensive research has revealed that EMT results in a decrease in E-cadherin expression, while N-cadherin and Vimentin expression levels increase [8]. As we know, the TGF-β signaling pathway plays an important role in regulating cellular activities, including cell migration, growth, and apoptosis. It is a widely prevalent pathway in various organisms. In humans, the pathway comprises 33 TGF distinct β family proteins, among which are 3 TGF-β (TGF-β1/2/3), 10 BMP, and 11 GDF [9, 10]. It is worth noting that this pathway is highly conserved across species and is vital to many biological processes. The Smad protein is a crucial molecule involved in signal transduction downstream of the TGF-β family receptor. Through Smad signal transduction, TGF-β enhances the movement and migration abilities of epithelial cells during EMT. In the TGF-β/Smad pathway, TGF-β forms a heteromeric complex with TGF-β type I receptor (TGFβR1) and TGF-β type II receptor (TGFβR2). This complex activates and phosphorylates Smad2 and Smad3, which then form a trimeric complex with Smad4. Therefore, E-cadherin is down-regulated, while N-cadherin and Vimentin are up-regulated [11–13]. TGFβR1, an indispensable upstream receptor in the TGF-β/Smad signaling pathway, plays an important role [14].

OLFM2 is a glycoprotein that is secreted and belongs to the protein family containing olfactomedin domains. In mammals, there are 13 members of this family, which is further divided into seven subfamilies [15, 16]. OLFM2 is involved in the induction of smooth muscle differentiation by the TGF-β and regulates the phenotypic switch of vascular smooth muscle cells.This is crucial in vascular remodeling and repair [17, 18]. Smooth muscle is an essential layer in blood vessels and helps maintain vascular tone. It is primarily composed of vascular smooth muscle cells [18]. Diseases are often associated with dysfunction of these cells. Furthermore, angiogenesis is critical in tumor infiltration and metastasis [19, 20]. Studies suggest that OLFM2’s involvement in cell adhesion and differentiation may be linked to cancer [15]. OLFM2 can inhibit distant metastasis of hepatocellular carcinoma cells and is associated with vascular invasion [21]. Therefore, it is hypothesized that OLFM2 may have a connection with CRC metastasis.

Currently, this study aims to investigate the function of OLFM2 in regulating the metastasis progression of CRC through the TGF-β/Smad pathway. By doing so, it seeks to establish a theoretical foundation for the diagnosis and treatment of CRC.

## Materials and methods

### Database bioinformatics analysis

Download colorectal cancer RNA sequencing transcriptome and clinical data from The Cancer Genome Atlas (TCGA) database. The R limma package was used for difference analysis, and the survival package was used for survival analysis, drawing the receiver operating characteristic (ROC) curve, drawing the nomogram, drawing the forest map, etc. The ggpubr, vioplot, ggExtra, and ggplot2 packages were used for tumor microenvironment analysis, immune infiltration analysis, drug sensitivity analysis, and immunotherapy analysis.

### Tissue sample collection

This study protocol was approved by the Ethics Committee of the Affiliated Hospital of North Sichuan Medical College. We obtained cancer tissues and adjacent normal tissues from 8 patients with CRC from the Affiliated Hospital of North Sichuan Medical College. Patient information is shown in Table 1.The patients did not undergo any treatment before surgery. All tissues were obtained within half an hour after surgery, transported at low temperature in liquid nitrogen, and frozen in liquid nitrogen for future use. Before the surgery, each patient was informed about this experiment, signed a notice.

**Table 1:** Patient information

Patient number	Sex	Age (years)	TNM stage	Mutation
1	Male	78	T2N1M0	KRAS
2	Male	72	T3N2M0	KRAS
3	Male	62	T2N1M0	KRAS
4	Female	68	T2N0M0	KRAS
5	Male	59	T4N2M0	KRAS
6	Female	79	T3N1M0	KRAS
7	Female	73	T4aN2M0	KRAS
8	Male	71	T2aN0M0	KRAS

### Cell culture

Our research group utilized the following cell lines: HCT15, SW620, and SW480, all of which were acquired from the cell bank of the esteemed Chinese Academy of Sciences. We employed a blend of RPMI 1640 (VivaCell Biosciences., China) and 10% fetal bovine serum (Inner Mongolia Opcel Biotechnology Co., Ltd. China) to cultivate the cells under sterile conditions in a 37 °C, 5% CO_2_ incubator.

### Cell transfection

The OLFM2-specific small interfering RNA (siRNA) was purchased from Shanghai IBS Biological Technology Co., Ltd. The OLFM2 overexpression plasmid was provided by Shanghai Genechem Co.,Ltd. The logarithmically growing colon cancer cells were seeded into a six-well plate and transfected with Lipo8000™ transfection reagent (Beyotime, China) once the cell density reached 70-80%. According to the instructions, Opti-MEM serum-free medium was mixed with siRNA or plasmid, and Lipo8000™ transfection reagent was added (incubate at normal temperature for 20 min when transfecting siRNA). Then, the mixture was inoculated in a six-well plate and grown in a cell incubator for 48 h. Finally, follow-up experiments were performed, including qRT-PCR, western blotting, cell proliferation, cell migration and invasion, cell scratch assays, etc.

### Real-time fluorescence quantitative polymerase chain reaction (qRT-PCR)

In accordance with the manufacturer’s instructions, an Oriscience Prime RNA Extraction Kit (Oriscience, China) was used to obtain total RNA from colon cancer cells and colorectal cancer tissues. The obtained total RNA was reverse transcribed into cDNA using the BeyoRT™ Q First Strand cDNA Synthesis Kit (Beyotime, China) according to the instructions. Finally, qRT-PCR was performed using BeyoFast™ SYBR Green qPCR Mix (2X) (Beyotime, China) on the LightCycler 96 (Roche, Switzerland). The internal reference we use is GAPDH. The target gene RNA was obtained by 2-ΔΔCt algorithm. The primer sequence is GAPDH-F GGAGTCCACTGGCGTCTTCA GAPDH-R GTCATGAGTCCTTCCACACGATACC OLFM2-F TCCTTGAGTTGCGGACGTATC OLFM2-R GCCGGAGAGATTCCTCACC.

### Western blotting

According to the instructions, PMSF and RIPA tissue / cell lysates (Solarbio, China) were added to cells or tissues, lysed on ice for 20 min, and then centrifuged for 15 min (4 ° C, 12,000 rpm). Then, the protein concentration of the supernatant was measured using the BCA concentration kit (Beyotime, China). Finally, SDS-PAGE protein loading buffer (5X) (Beyotime, China) was added, and then the protein was denatured by metal bath for 10 min. After the protein extraction was completed and the concentration was determined, an appropriate amount of sample protein was added to the electrophoresis gel for electrophoresis separation, and then transferred to the PVDF membrane (Solarbio, China). PVDF membranes were sealed with a rapid blocking solution and incubated overnight with primary antibody in a refrigerator at 4 ° C, and the next day with secondary antibody (1:20000, HA1024; HUABIO) were incubated at room temperature for 60 min. In the last step, we used the Ori Ultrasensitive ECL Kit (Oriscience, China) to observe the bands. The antibodies used were OLFM2 (1:1000, ab154196; Abcam), GAPDH (1:30000, SA30-01; HUABIO), E-cadherin (1:5000, ET1607-75; HUABIO), N-cadherin (1:1000,ET1607-37; HUABIO), Vimentin (1:20000,ET1610-39; HUABIO), TGF-βR1 (1:1000,PB1154; BOSTER), Phospho-Smad3 (1:1000,C25A9; CST), Phospho-Smad2 (1:1000, D27F4; CST).

### Cell proliferation assay

The proliferation of cells was measured using the CCK-8 kit (Oriscience, China). Cells were inoculated into 96-well plates at a density of 2 × 10^3^ cells / well. We added CCK-8 to each well at 0, 24, 48, and 72 h, then incubated it for an hour at 37 °C with 5% CO2.Finally, the absorbance values at 450 nm were measured using a microplate reader.

### Cell wound scratch assay

After transfecting cells with siRNA or overexpression plasmids for 48 h, transfer an appropriate amount of cells to a 6-well plate. When the cells reach a confluency of over 90%, initiate the scratch by creating a wound. Replace the serum-free medium and take microscope images at 0 and 48 h, respectively.

### Transwell assay

After transfecting cells with siRNA or overexpression plasmids for 48 h, perform cell counting. In migration experiments, first resuspend cells (5 × 10^4^) in 200 µl of serum-free medium. Then, seed the cell suspension into a transwell chamber, and finally place the chamber in a 24-well plate containing 500 µl of complete medium. In invasion experiments, seed cells (6 × 10^4^) into transwell chambers coated with an extracellular matrix gel (BD Bioscience). Incubate the 24-well plate in a cell culture incubator for 48 h under normal growth conditions. Remove the transwell chamber, wash it with PBS, fix it in 4% paraformaldehyde for half an hour, stain it with crystal violet solution for half an hour, and finally use a cotton swab to wipe off excess dye and non-migrated cells inside the chamber. After air-drying, select different fields of view for photography. After air-drying, take photographs of different fields of view.

### Statistical analysis

Perform graphic and data processing using R 4.1.2 (https://www.r-project.org/), GraphPad Prism 8.0.1 (GraphPad Software Inc., La Jolla, USA), and Image J 1.51 (National Institutes of Health, USA).The data is presented as mean ± standard deviation (SD). The results of two groups of data were processed by unpaired t-test. *P* < 0.05 indicates a statistically significant difference, except for other explanations (* *p* < 0.05, ** *p* < 0.01, *** *p* < 0.001).

### Ethics statement

Before conducting this experiment, our research obtained approval from the Ethics Committee of the Affiliated Hospital of North Sichuan Medical College (2023ER133-1), and it was conducted following the principles of the Helsinki Declaration.

## Results

### OLFM2 could be used as an independent prognostic factor

**Fig. 1:**
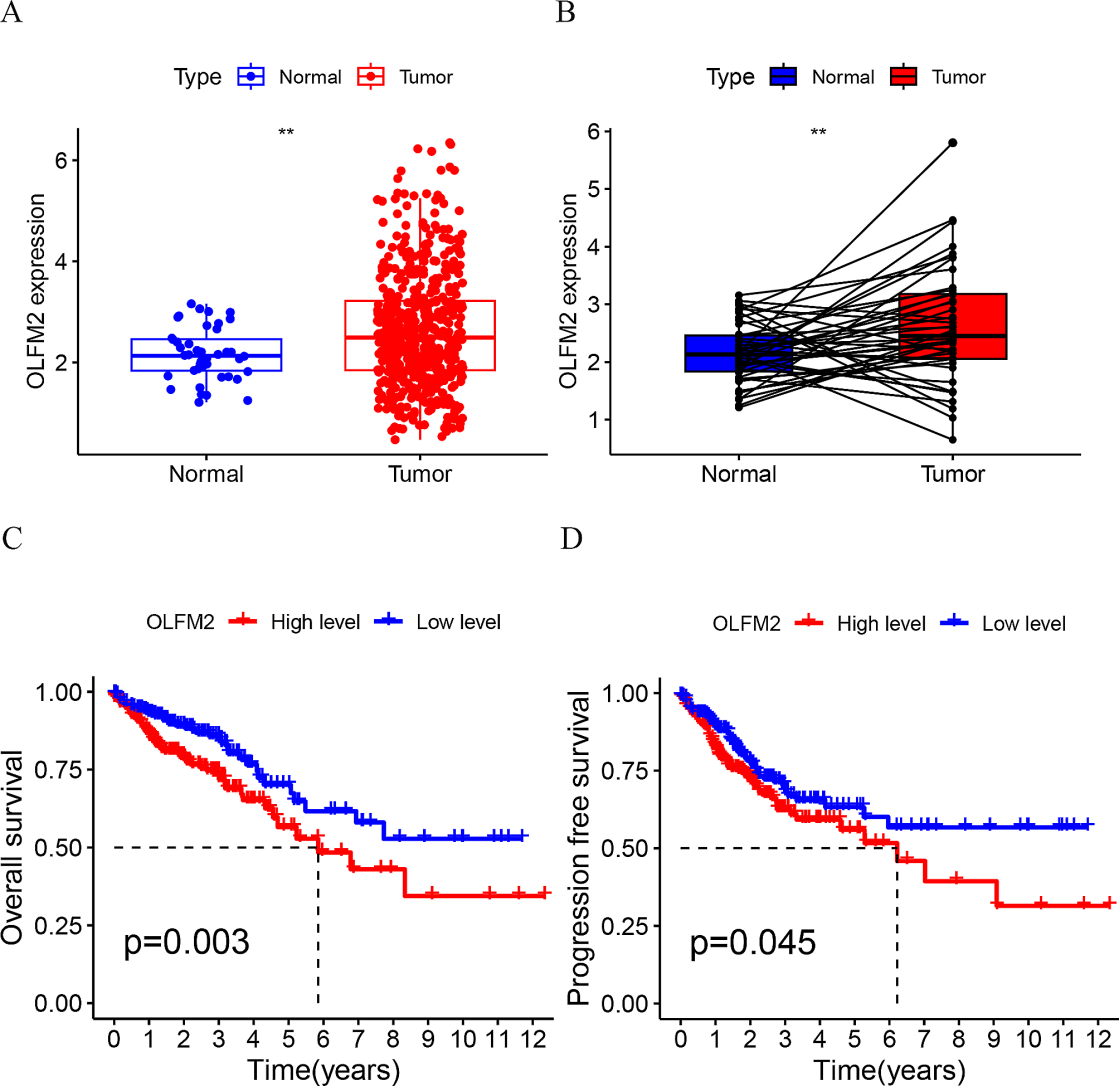
(**A**) The expression of OLFM2 in colorectal cancer using data from the TCGA database. (**B**) The OLFM2 expression between cancer tissues and adjacent tissues from patients. (**C**) The survival analysis curve of OLFM2. (**D**) Progression-free survival analysis of OLFM2

**Fig. 2:**
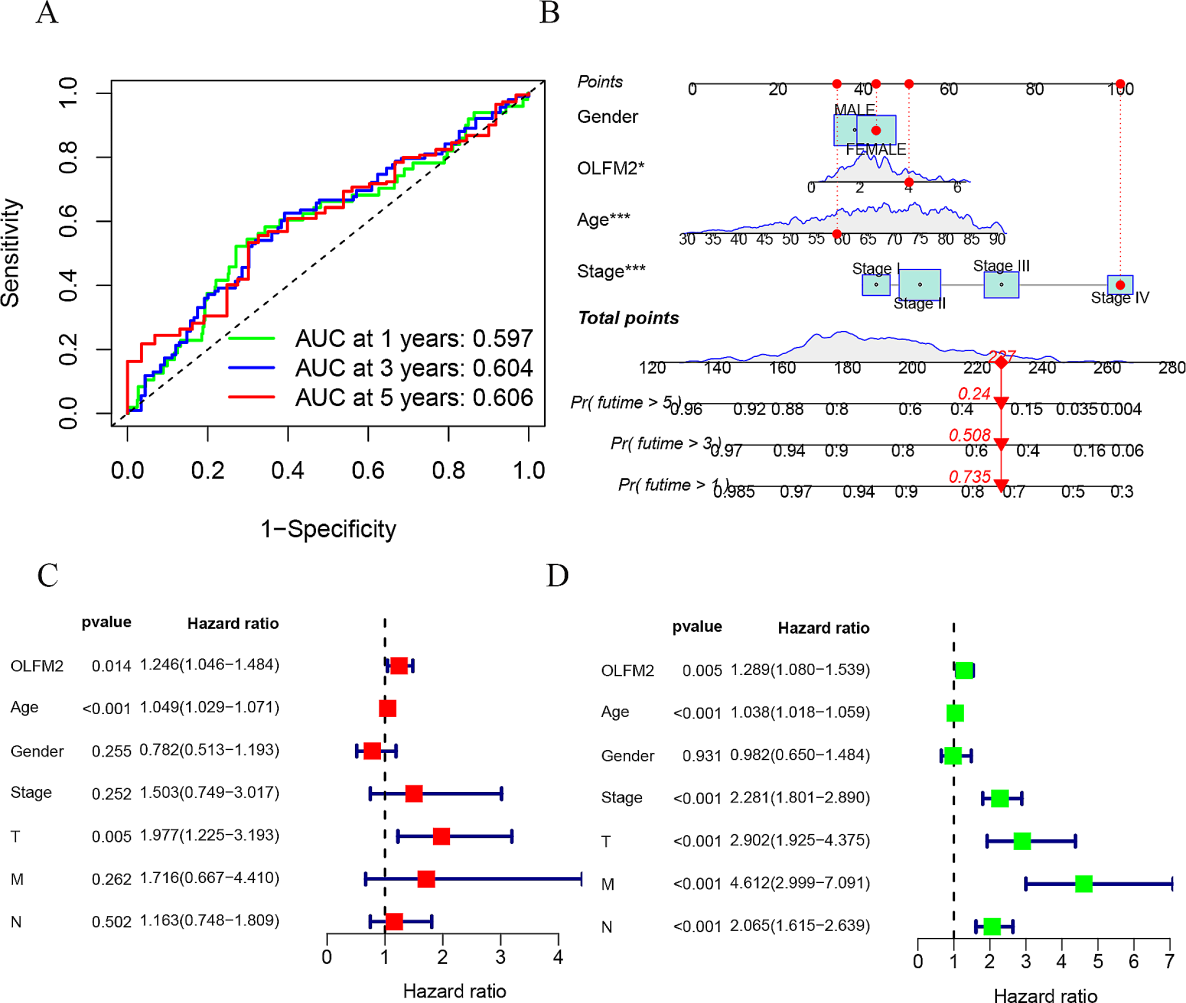
(**A**) The ROC curve of OLFM2 expression to predict the patient’s 1.3.5-year survival time. (**B**) A nomogram for predicting the 1.3.5-year survival rate of patients. (**C**) OLFM2 multivariate independent prognostic analysis. (**D**) OLFM2 univariate independent prognostic analysis

We obtained RNA sequence transcriptome data and clinical information from the TCGA database, comprising 568 cases of CRC and 44 cases of adjacent tissue. After performing data normalization, we extracted the expression matrix of OLFM2. As depicted in Fig. 1A and B, the mRNA expression of OLFM2 was significantly higher in the tumor tissue. Our subsequent survival analysis indicated that patients with low expression of OLFM2 had better survival rates than those with high expression (Fig. 1C and D). Additionally, we plotted a ROC curve based on the expression level of OLFM2, which accurately predicted 1-year, 3-year, and 5-year survival rates in CRC patients with accuracies of 0.597, 0.604, and 0.606, respectively (Fig. 2A). To develop a nomogram, we integrated patient age, gender, and stage, which revealed survival rates of 0.735, 0.508, and 0.24 for 1-year, 3-year, and 5-year survival, respectively (Fig. 2B). Lastly, both univariate and multivariate Cox regression analysis confirmed that OLFM2 may serve as an independent prognostic factor (Fig. 2C and D).

### Sensitivity of OLFM2 to immunotherapy and drug therapy

**Fig. 3:**
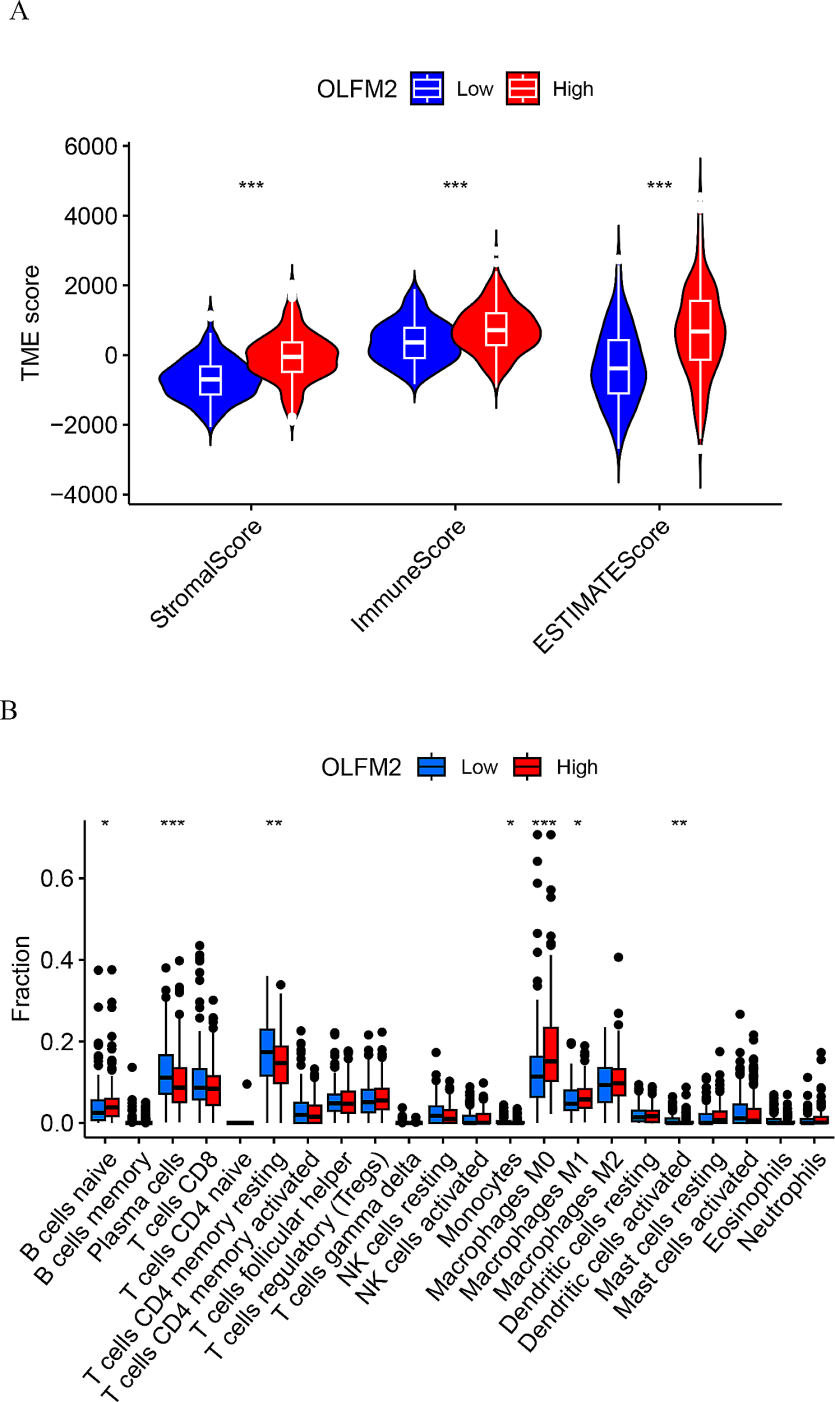
(**A**) Analysis of tumor microenvironment differences between OLFM2 high and low expression groups. (**B**) Differential analysis of immune cells in OLFM2 high and low expression groups

Within the tumor microenvironment, non-tumor components are primarily composed of immune cells and stromal cells. A comparison of high and low-expression groups of OLFM2 reveals varying stromal cell scores, immune cell scores, and comprehensive scores, as displayed in Fig. 3A. Notably, higher expression of OLFM2 correlates with increased content of stromal and immune cells. Further analysis of immune cell differences indicates that B cells naive, Plasma cells, T cells CD4 memory resting, Monocytes, Macrophages M0, Macrophages M1, and Dendritic cells activated were all distinct between the high and low expression groups of OLFM2, as depicted in Fig. 3B.

**Fig. 4:**
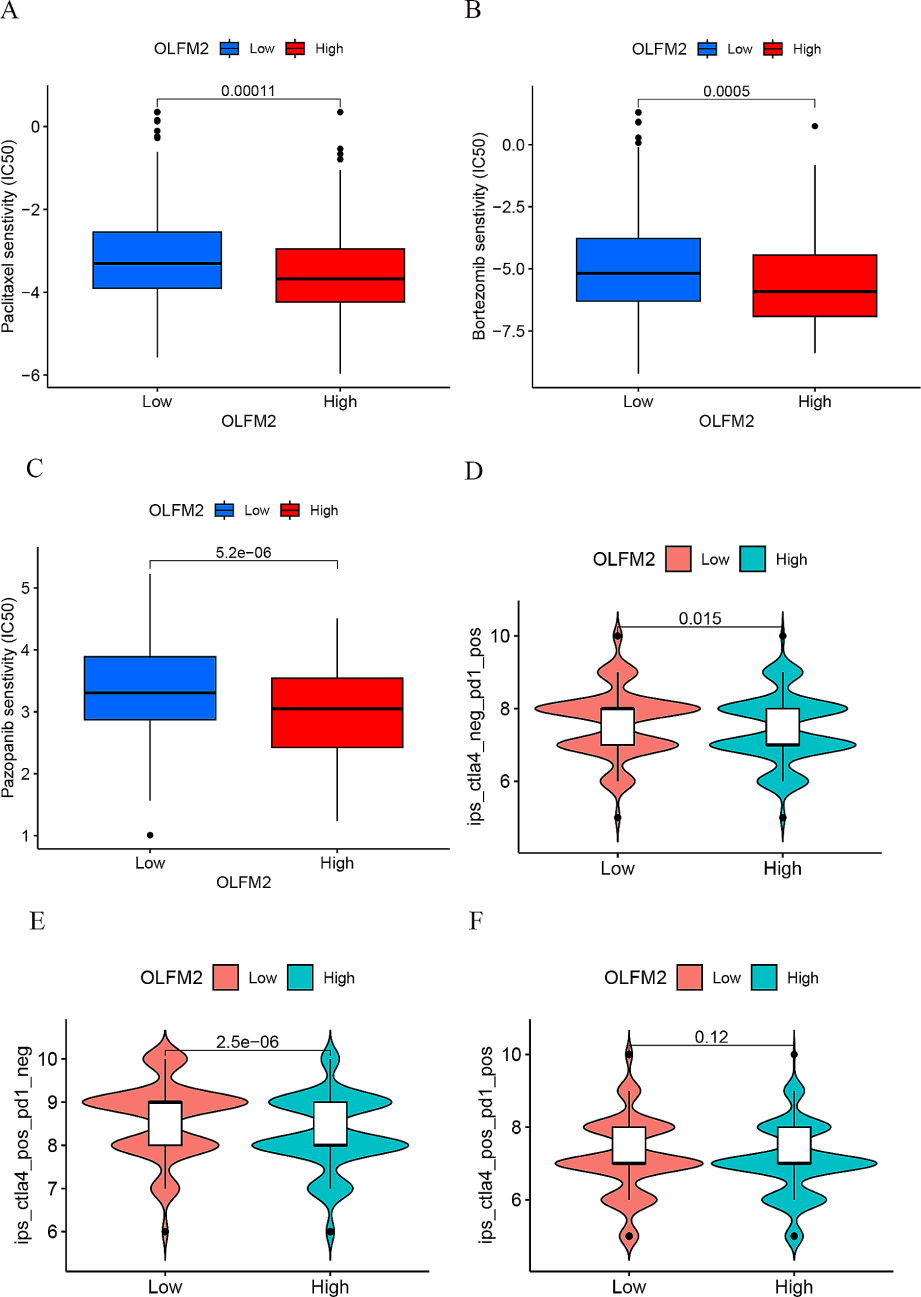
(**A–C**) Drug sensitivity analysis results of OLFM2 high and low expression groups. (**D–F**) Immunotherapy analysis results of OLFM2 high and low expression groups

Based on the drug sensitivity analysis, the OLFM2 high-expression group exhibited greater sensitivity to Paclitaxel, Bortezomib, and Pazopanib compared to the low-expression group (Fig. 4A-C). The high-expression group also demonstrated a more favorable response to chemotherapeutic drugs. Meanwhile, immunotherapy analysis revealed varying responses between the high and low expression groups of OLFM2. As demonstrated in Fig. 4D, the OLFM2 low expression group displayed a better response to anti-PD1 treatment. Similarly, the OLFM2 low expression group also exhibited an improved response to anti-CTLA4 treatment, as shown in Fig. 4E. However, there was no significant difference observed between OLFM2 high and low expression groups in response to anti-PD1 and anti-CTLA4 combination therapy (Fig. 4F).

### OLFM2 is highly expressed in CRC tissues

**Fig. 5:**
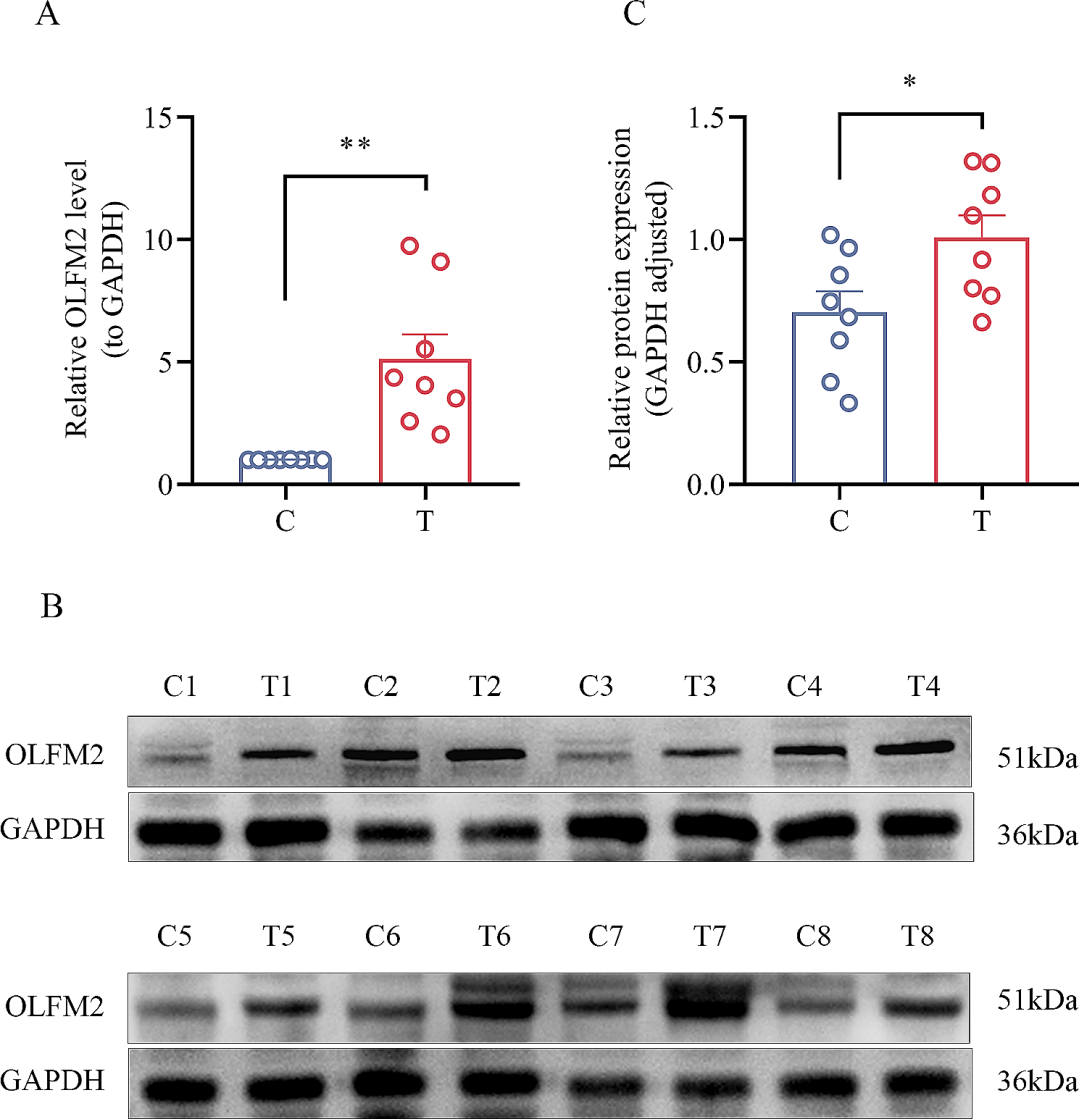
(**A**) Results of qRT-PCR for OLFM2 in 8 pairs of tissues (T and C indicate CRC and adjacent tissues, respectively). (**B**, **C**) Western blotting for OLFM2 in eight pairs of clinical tissues

Following the collection of colorectal cancer (CRC) tissue samples, we conducted qRT-PCR and western blotting analyses on eight pairs of tissue specimens to examine the expression of OLFM2 mRNA and protein. Our results revealed that CRC tissues exhibited significantly higher levels of OLFM2 mRNA and protein compared to adjacent non-cancerous tissues (Fig. 5).

### OLFM2 enhances the proliferation of colon cancer cells

**Fig. 6:**
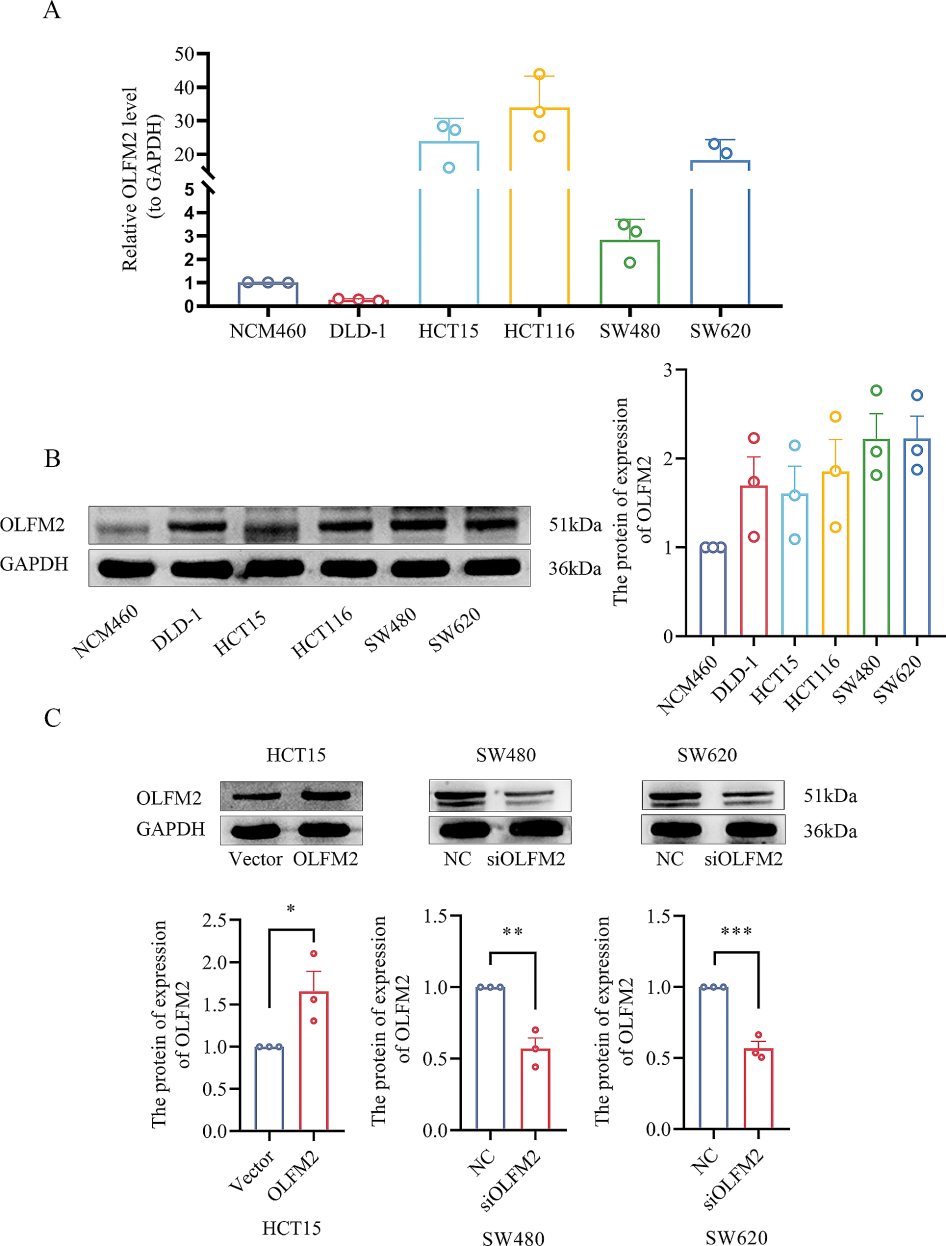
(**A**) The qRT-PCR results of OLFM2 in various cell lines. (**B**) Western blotting results of OLFM2 protein in each cell line. (**C**) The expression of OLFM2 protein after transfection of plasmid and siRNA (Vector refers to the empty control group, OLFM2 refers to the plasmid overexpression group, NC refers to the negative control group, siOLFM2 refers to siRNA knockdown group)

We conducted a comparison of mRNA and protein levels of OLFM2 in cells from NCM460, HCT15, HCT116, DLD-1, SW480, and SW620 via qRT-PCR and western blotting (Fig. 6A and B). Based on the results, we selected HCT15 cells to establish an overexpression cell line as shown in Fig. 6C. The OLFM2 protein was significantly upregulated in the overexpression group. Additionally, we chose SW480 and SW620 cells to establish knockdown cell lines, which showed significant downregulation of the OLFM2 protein in the knockdown group (Fig. 6C).

**Fig. 7:**
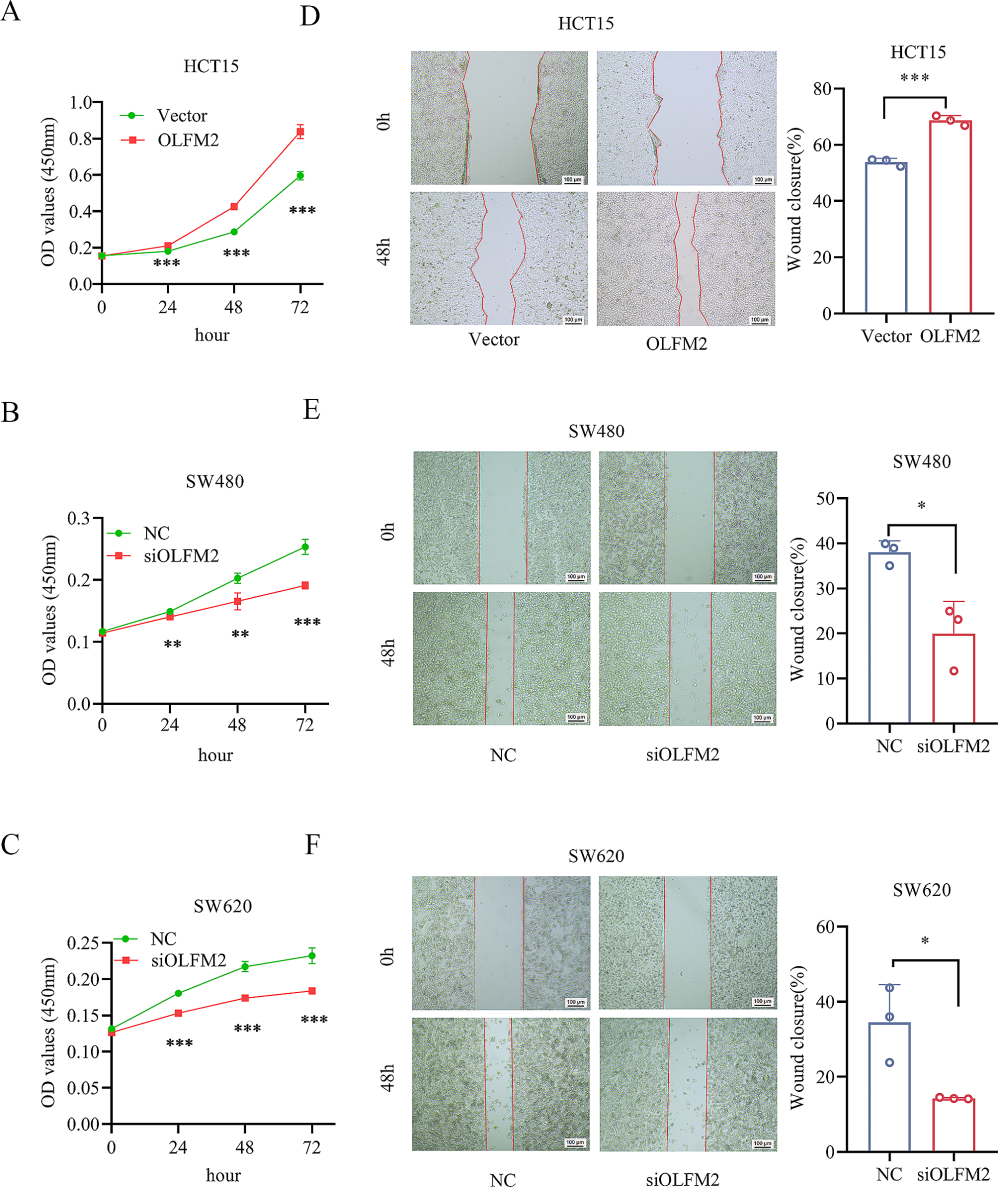
(**A**) The growth curve of HCT15 cells in the OLFM2 group and Vector group. (**B**) The growth curve of SW480 cells in the siOLFM2 group and NC group. (**C**) The growth curve of SW620 cells in the siOLFM2 group and NC group. (**D**) Scratch results of HCT15 cells in the OLFM2 group and Vector group at 0 and 48 h. (**E**) Scratch results of SW480 cells in siOLFM2 group and NC group at 0 and 48 h. (**F**) Scratch results of SW620 cells in the siOLFM2 group and NC group at 0 and 48 h

To analyze the influence of OLFM2 on CRC cell growth, we conducted a CCK-8 proliferation assay and generated a growth curve for the cells. In Fig. 7A-C, the results illustrate that the upregulation of OLFM2 resulted in heightened growth for HCT15 cells, whereas the downregulation of OLFM2 caused a decline in cell growth for groups SW480 and SW620, compared with the control group.

### OLFM2 promotes the migration and invasion of CRC cells

**Fig. 8:**
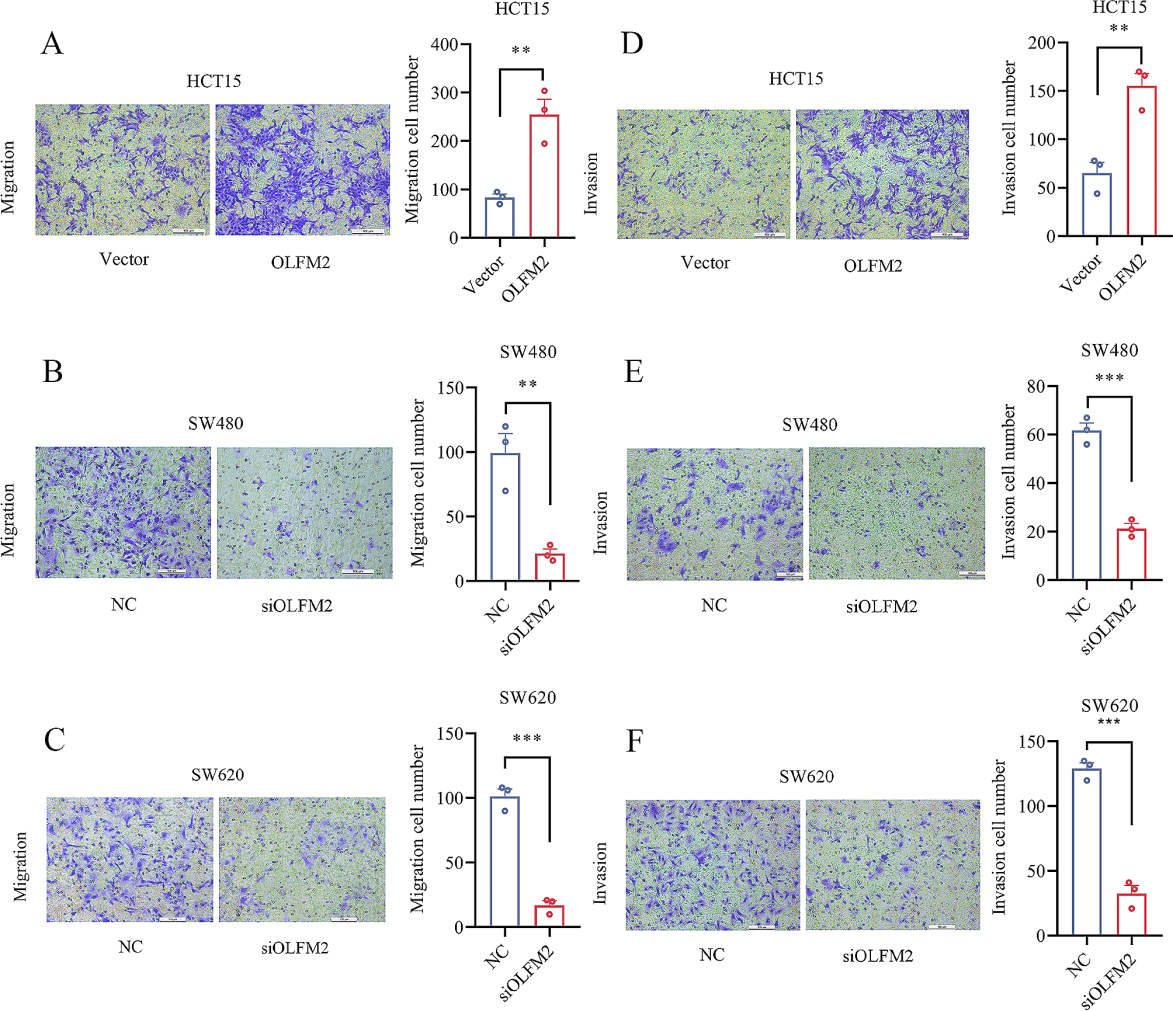
(**A**, **D**) The results of migration and invasion of HCT15 cells in the OLFM2 group and Vector group. (**B**, **E**) The results of migration and invasion of SW480 cells in the siOLFM2 group and NC group. (**C**, **F**) The results of migration and invasion of SW620 cells in the siOLFM2 group and NC group

Our study focused on the impact of OLFM2 on CRC cell migration and invasion, which we assessed through cell scratch and transwell assay. Our findings revealed that the HCT15 overexpression group had a higher rate of wound closure 48 h after cell scratching compared to the control group (Fig. 7D). Conversely, the knockdown groups showed a lower rate of wound closure (Fig. 7E and F). Similarly, in the transwell migration assay, the HCT15 overexpression group demonstrated a greater number of migrated cells 48 h later than the control group (Fig. 8A), whereas the knockdown groups exhibited a reduced number of migrated cells (Fig. 8B and C). To assess cell invasion ability, we conducted a transwell invasion assay with matrix gel added to the chamber. Our results showed that the HCT15 overexpression group had a significantly stronger invasion ability than the control group, while the knockdown groups displayed a significantly weaker invasion ability than the control group (Fig. 8D, E, and F). Overall, our findings suggest that OLFM2 enhances the migration and invasion abilities of CRC cells.

### OLFM2 promotes the EMT process of CRC cells

**Fig. 9:**
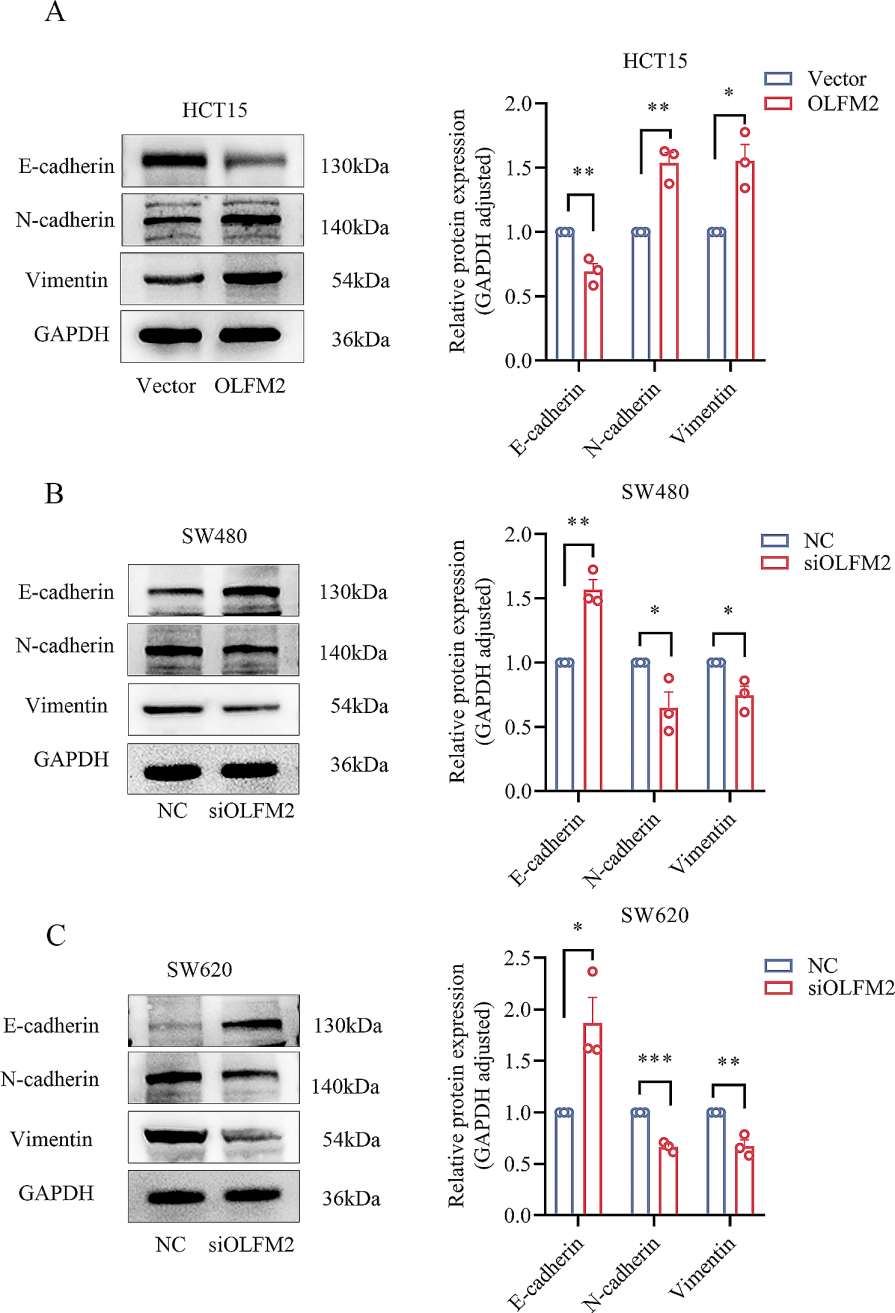
(**A**) The expression of E-cadherin, N-cadherin, and Vimentin protein in HCT15 cells of the OLFM2 group and Vector group. (**B**) The expression of E-cadherin, N-cadherin, and Vimentin protein in siOLFM2 group and NC group of SW480 cells. (**C**) The expression of E-cadherin, N-cadherin, and Vimentin protein in siOLFM2 group and NC group of SW620 cells

As previously discussed, the process of EMT in cancer cells involves a decrease in the expression of E-cadherin and an increase in the expression of N-cadherin and Vimentin. In HCT15 cells, overexpression of OLFM2 leads to a decrease in E-cadherin expression and an increase in N-cadherin and Vimentin expression (Fig. 9A). Conversely, when OLFM2 is knocked down in cells SW480 and SW620, there is an increase in E-cadherin expression and a decrease in N-cadherin and Vimentin expression (Fig. 9B and C).These findings indicate that OLFM2 plays an important role in promoting EMT in colon cancer cells.

### OLFM2 activates the TGF-β/Smad signaling pathway in CRC cells

**Fig. 10:**
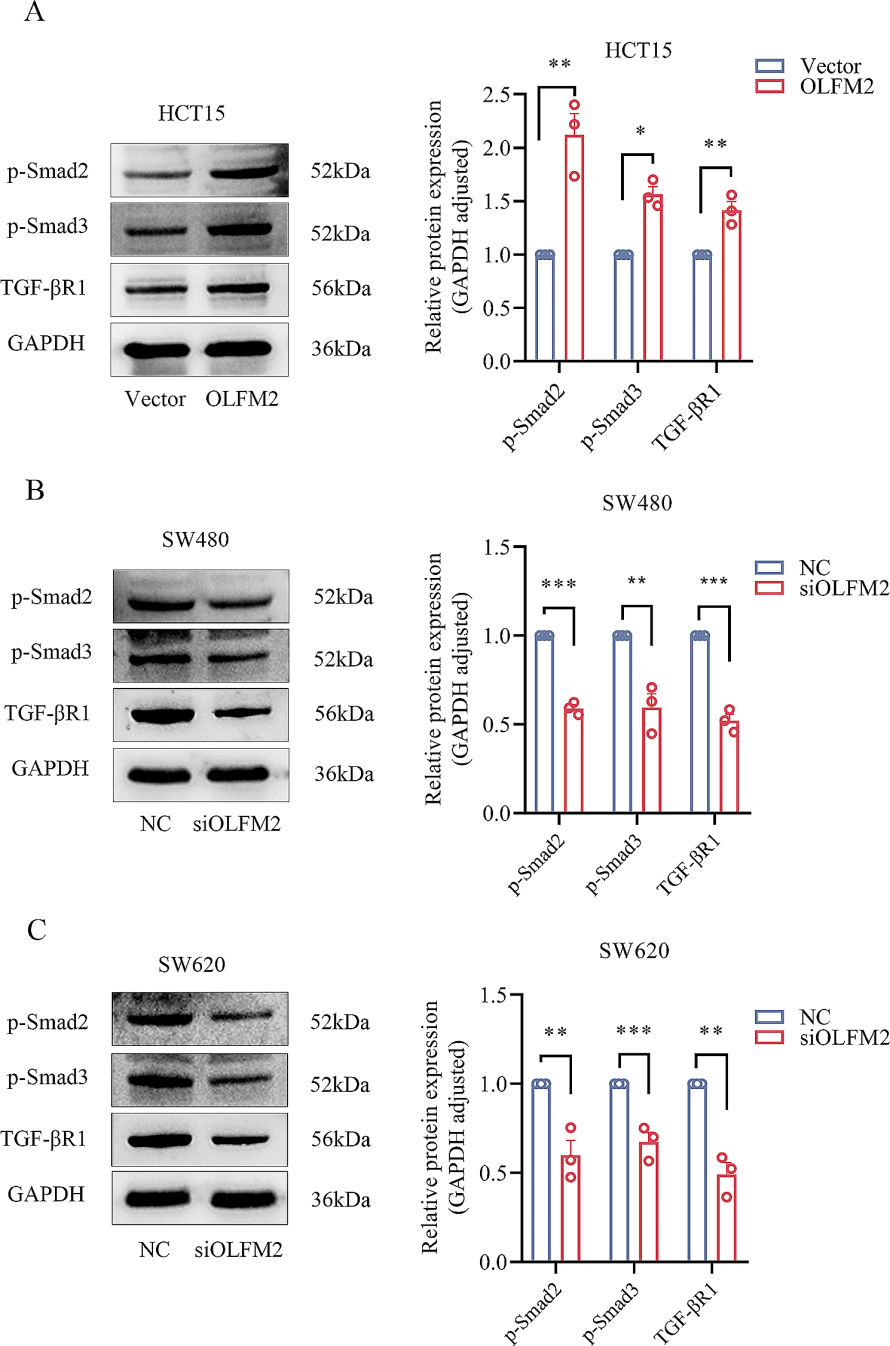
(**A**) The expression of TGF-βR1, p-Smad2, and p-Smad3 protein in HCT15 cells in the OLFM2 group and Vector group. (**B**) The expression of TGF-βR1, p-Smad2, and p-Smad3 protein in the siOLFM2 group and NC group in SW480 cells. (**C**) The expression of TGF-βR1, p-Smad2, and p-Smad3 protein in the siOLFM2 group and NC group in SW480 cells

In our research, we hypothesized that OLFM2 plays a role in the development of CRC cells via the TGF-β signaling pathway. To achieve this, we investigated how OLFM2 affects the classical TGF-β/Smad signaling transduction. Our findings showed that when OLFM2 was overexpressed, TGF-βR1, p-Smad2, and p-Smad3 expression in HCT15 cells increased (Fig. 10A). Conversely, when OLFM2 was knocked down, the expression of TGF-βR1, p-Smad2, and p-Smad3 in SW480 and SW620 cells decreased (Fig. 10B and C). Therefore, based on these results, we determined that OLFM2 can promote colorectal cancer EMT, migration, and invasion through the TGF-β/Smad signaling pathway.

## Discussion

Our experiment highlights OLFM2 as a potential oncogene in CRC. Our study reveals that the OLFM2 gene is significantly upregulated in CRC patients’ tissues, and its overexpression is linked to poor prognosis. Although OLFM2 has been seldom reported in other tumors, its mechanisms in colorectal cancer metastasis and growth remain unknown. Our data indicates that OLFM2 triggers CRC cell metastasis by activating the TGF-β/Smad signaling pathway in vitro.

Epithelial cells undergo a series of processes known as EMT, whereby they lose their polarity, intercellular connections, and detachment from the basement membrane. This transformation into a mesenchymal phenotype is facilitated by specific procedures, which enhance the cells’ migration ability and enable them to acquire the ability to migrate and invade, a crucial factor in tumor metastasis and invasion according to research [22–24]. E-cadherin, a regulator of various cellular functions such as cell polarity, cell proliferation and differentiation, cell migration, and movement, is down-regulated during EMT. This inhibition prevents tumor development and dispersion while promoting tumor cell aggregation by providing an adhesive function [25, 26]. Another crucial EMT indicator is N-cadherin, which is significantly up-regulated during EMT and tumor progression. It promotes tumor cell growth, migration, and angiogenesis while protecting vascular integrity [27–29]. Vimentin, a typical EMT marker, is a vital regulator of cell movement that plays a significant role in maintaining cell integrity and enhancing cell anti-stress ability. Its increased expression is closely related to tumor proliferation, metastasis, and poor prognosis in various tumors [30–32]. It has also been reported that EMT plays an important role in the classification of colorectal cancer based on immunohistochemical detection of EMT-related markers [33, 34]. According to our study, the knockdown of OLFM2 resulted in increased expression of E-cadherin, while the expression of N-cadherin and Vimentin decreased. Conversely, overexpression of OLFM2 led to a decrease in E-cadherin expression and an increase in N-cadherin and Vimentin expression. These results indicate that OLFM2 plays a role in promoting EMT. However, the exact mechanism by which OLFM2 mediates the EMT process still requires further investigation.

Recent reports have shown that the TGF-β/Smad signaling pathway plays an important role in regulating tumor growth, movement, and invasion [8]. There is also evidence that this pathway is involved in the process of cancer cell EMT [35, 36], where TGF-β suppresses the expression of E-cadherin by activating SNAIL, ZEB, and TWIST, hence initiating EMT in cancer cells [37, 38]. TGF-β activates the receptor in the TGF-β/Smad signaling pathway, leading to the phosphorylation and activation of Smad2 and Smad3, which then triggers the inhibition or activation of target gene transcription [39]. In our study, we focused on TGFβR1, which is a key receptor upstream in this pathway [14]. Our findings indicate that OLFM2 knockdown reduced the expression of TGFβR1, leading to a decrease in the expression of downstream phosphorylated Smad2 and Smad3. Conversely, the overexpression of OLFM2 results in the upregulation of TGFβR1, leading to the upregulation of downstream phosphorylated Smad2 and Smad3. Therefore, we conclude that OLFM2 promotes EMT, migration, and invasion of CRC cells through the TGF-β/Smad signaling pathway.

In our study, we verified the role of OLFM2 in colon cancer cells through in vitro experiments. Our results showed that overexpression of OLFM2 in HCT15 cells led to enhanced cell migration, invasion, and proliferation, while knockdown of OLFM2 in cells SW480 and SW620 showed the opposite effects. These findings suggest that OLFM2 could be a potential target for CRC treatment. Additionally, we preliminarily verified that OLFM2 is closely related to the TGF-β pathway and EMT. However, our study has some limitations. Firstly, we did not conduct any in vivo experiments, and secondly, the number of clinical tissue specimens we analyzed was too small. Microsatellite instability (MSI) status, RAS gene, and BRAF gene mutations are recognized as the main markers affecting the treatment of CRC patients, and KRAS mutation is one of the most common mutations in CRC [40, 41]. Different mutation states have different choices for targeted therapy [42]. Since the cell line used in our study has a KRAS mutation, this is a limitation of our study.We hope that future research will address these limitations and explore the mechanisms involved at a deeper level.

## Conclusion

In summary,we suggested that OLFM2 may be a novel oncogene in CRC. Additionally, we also found that OLFM2 can promote EMT, migration, and invasion of CRC cells via the TGF-β/Smad pathway. These findings indicate that OLFM2 could be a promising target for the diagnosis and management of CRC.

### Electronic supplementary material

Below is the link to the electronic supplementary material.

Supplementary Material 1

## Data Availability

Data analyzed in this study are available in the following public databases: TCGA (https://portal.gdc.cancer.gov/repository).
