# Supplementary material for: OLFM2 promotes epithelial-mesenchymal transition, migration, and invasion in colorectal cancer through the TGF-β/Smad signaling pathway
Source: BMC Cancer. 2024 Feb 13;24:204. doi: 10.1186/s12885-024-11925-3 (PMC10865519; doi:10.1186/s12885-024-11925-3)

Eight pairs of tissue western blotting original images of Fig.5B

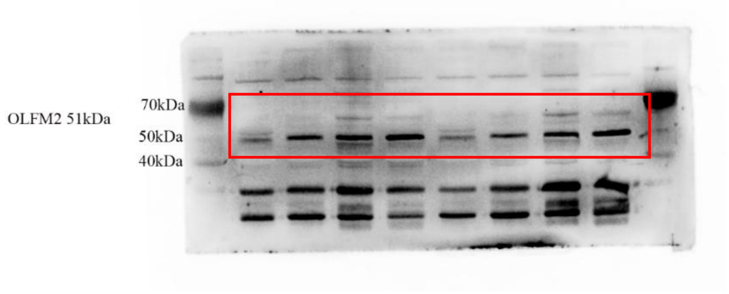

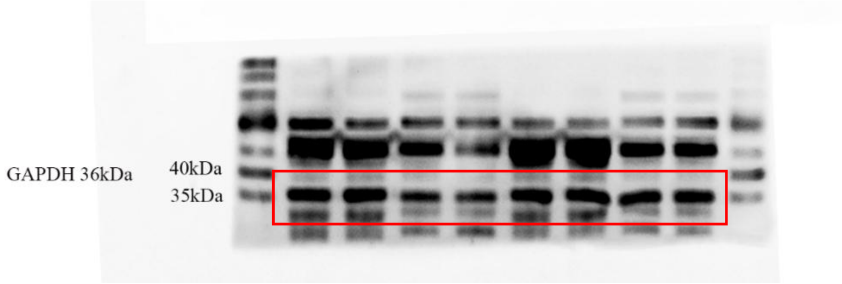

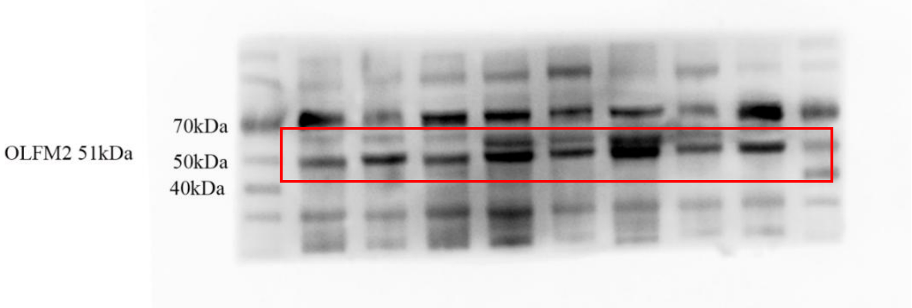

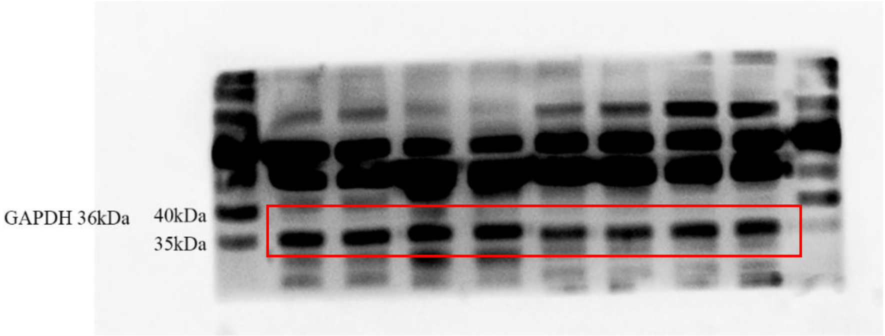

Figure 6B : Cell screening western blotting original images

Repeat1

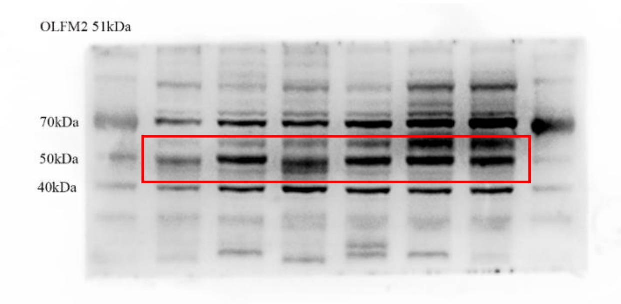

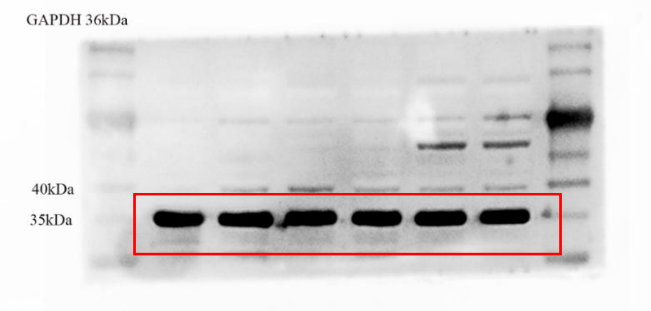

Repeat2

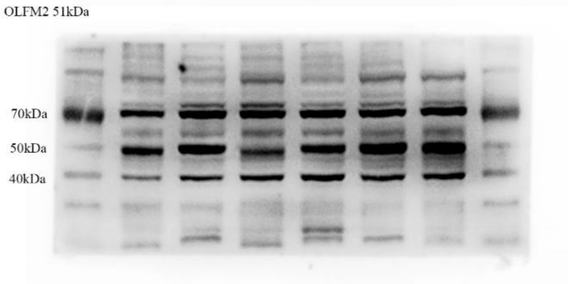

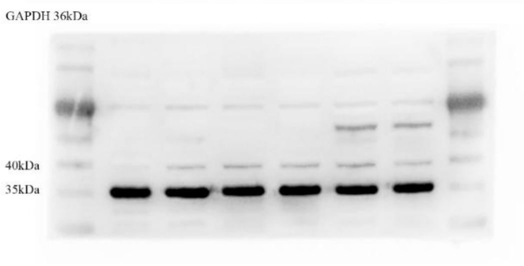

Repeat3

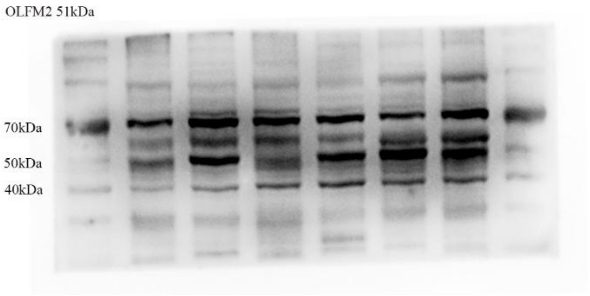

GAPDH 36kDa

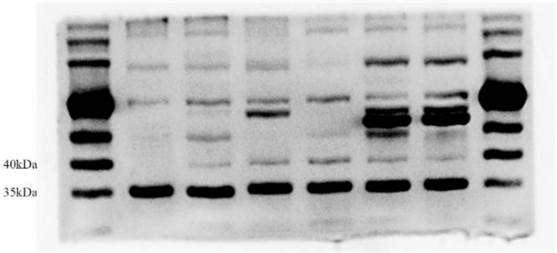

Fig6C:

HCT15 overexpression western blotting original images

Repeat1

OLFM2 51kDa

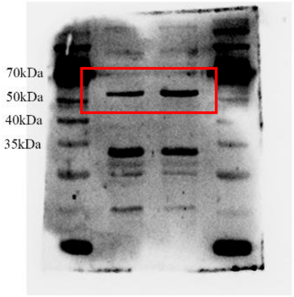

GAPDH 36kDa

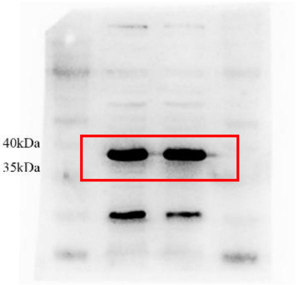

Repeat2

OLFM2 51kDa

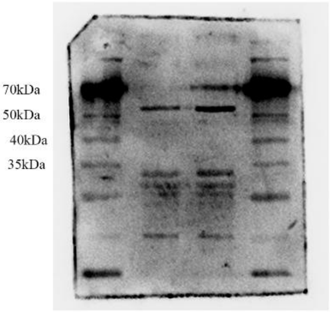

GAPDH 36kDa

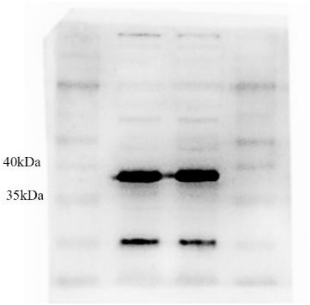

Repeat3

OLFM2 51kDa

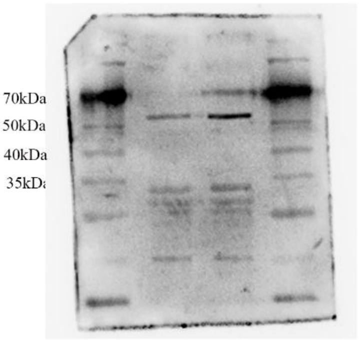

GAPDH 36kDa

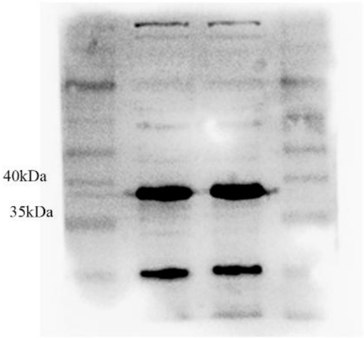

SW480 knockdown western blotting original images

Repeat1

OLFM2 51kDa

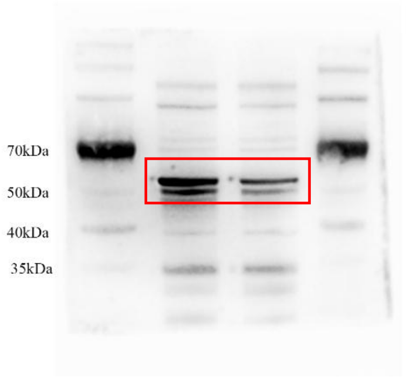

GAPDH 36kDa

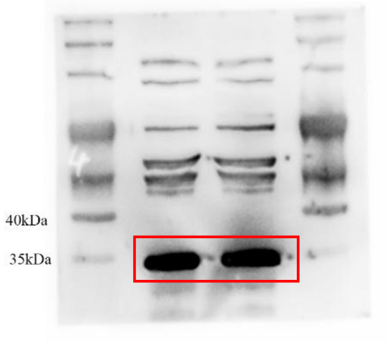

Repeat2

OLFM2 51kDa

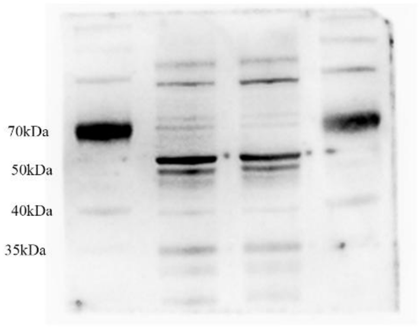

GAPDH 36kDa

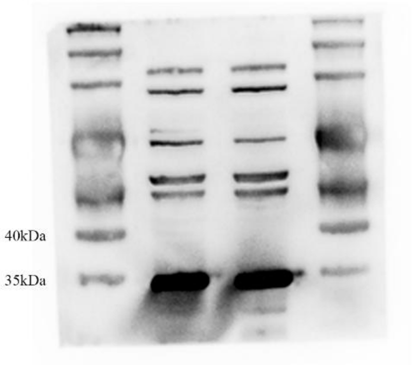

Repeat3

OLFM2 51kDa

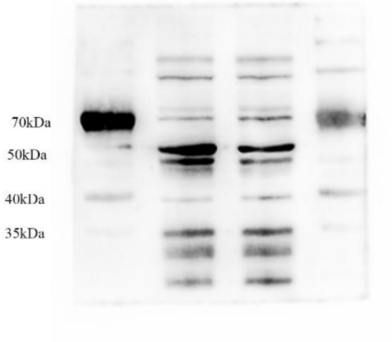

GAPDH 36kDa

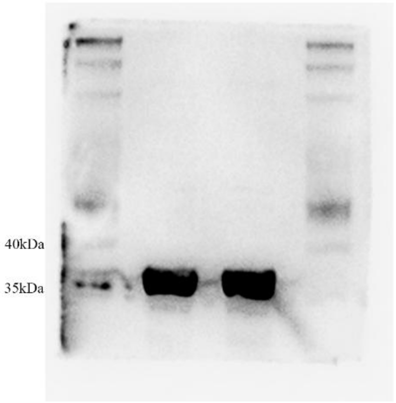

SW620 knockdown western blotting original images

Repeat1

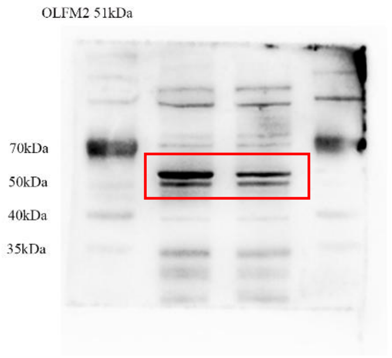

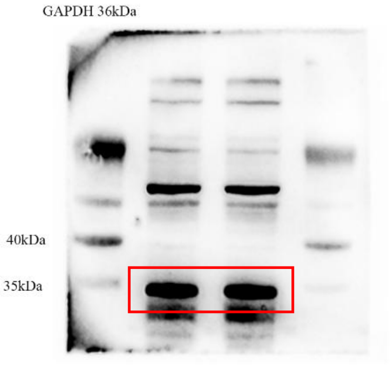

Repeat2

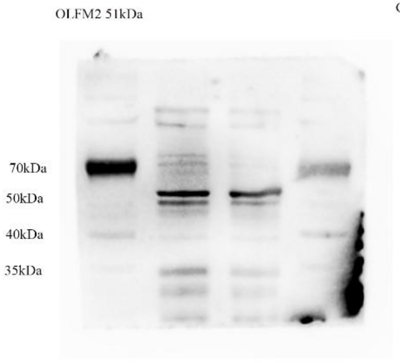

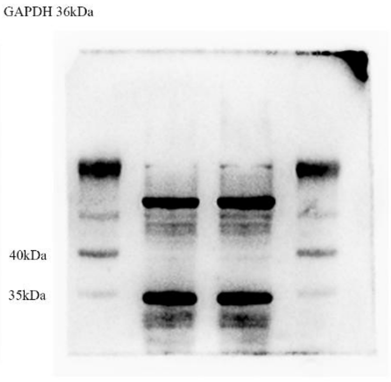

Repeat3

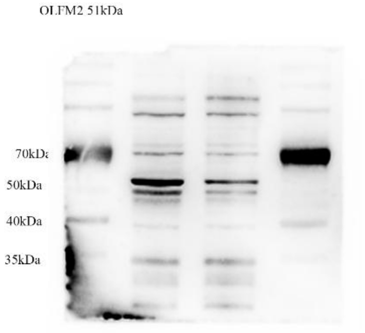

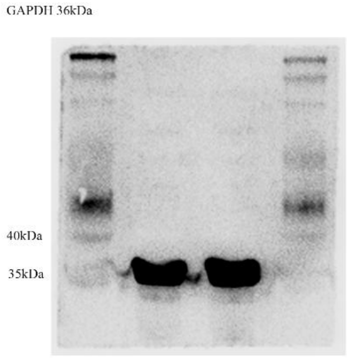

Original western blotting images for Figure 9A (E-cadherin,N-cadherin,Vimentin,GADPH)-HCT15

Repeat1

E-cadherin 130kDa

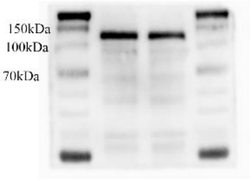

GAPDH 36kDa

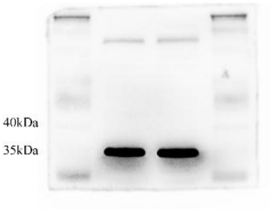

Repeat2

E-cadherin 130kDa

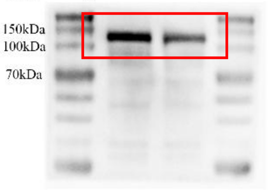

GAPDH 36kDa

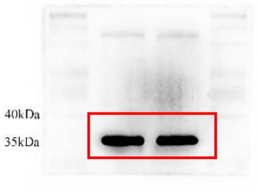

Repeat3

E-cadherin 130kDa

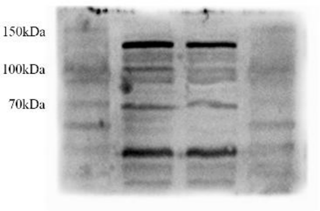

GAPDH 36kDa

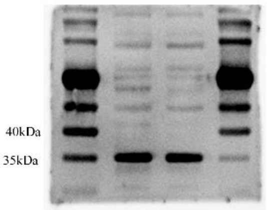

Repeat1

N-cadherin 140kDa

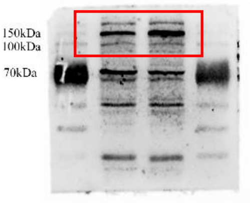

GAPDH 36kDa

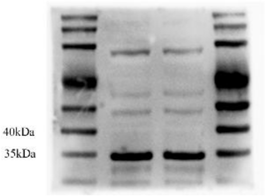

Repeat2

N-cadherin 140kDa

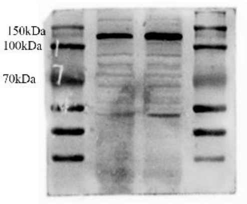

GAPDH 36kDa

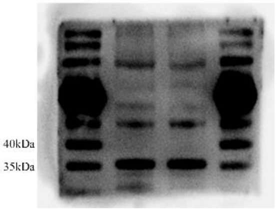

Repeat3

N-cadherin 140kDa

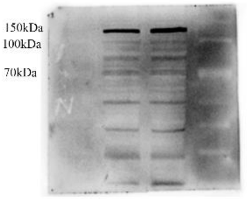

GAPDH 36kDa

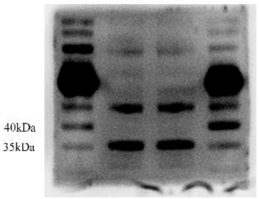

Repeat1

Vimentin 54kDa

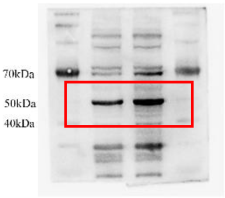

GAPDH 36kDa

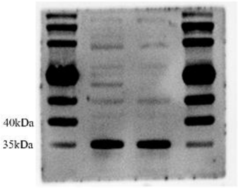

Repeat2

Vimentin 54kDa

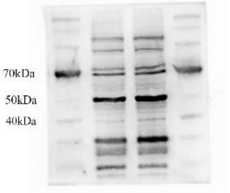

GAPDH 36kDa

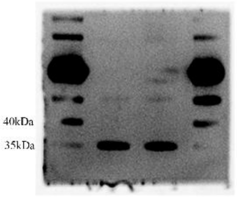

Repeat3

Vimentin 54kDa

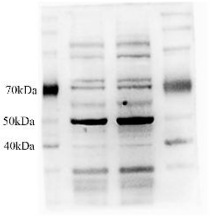

GAPDH 36kDa

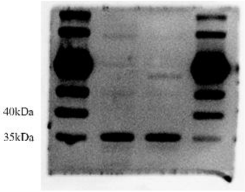

Original western blotting images for Figure 9B (E-cadherin,N-cadherin,Vimentin,GADPH)-SW480

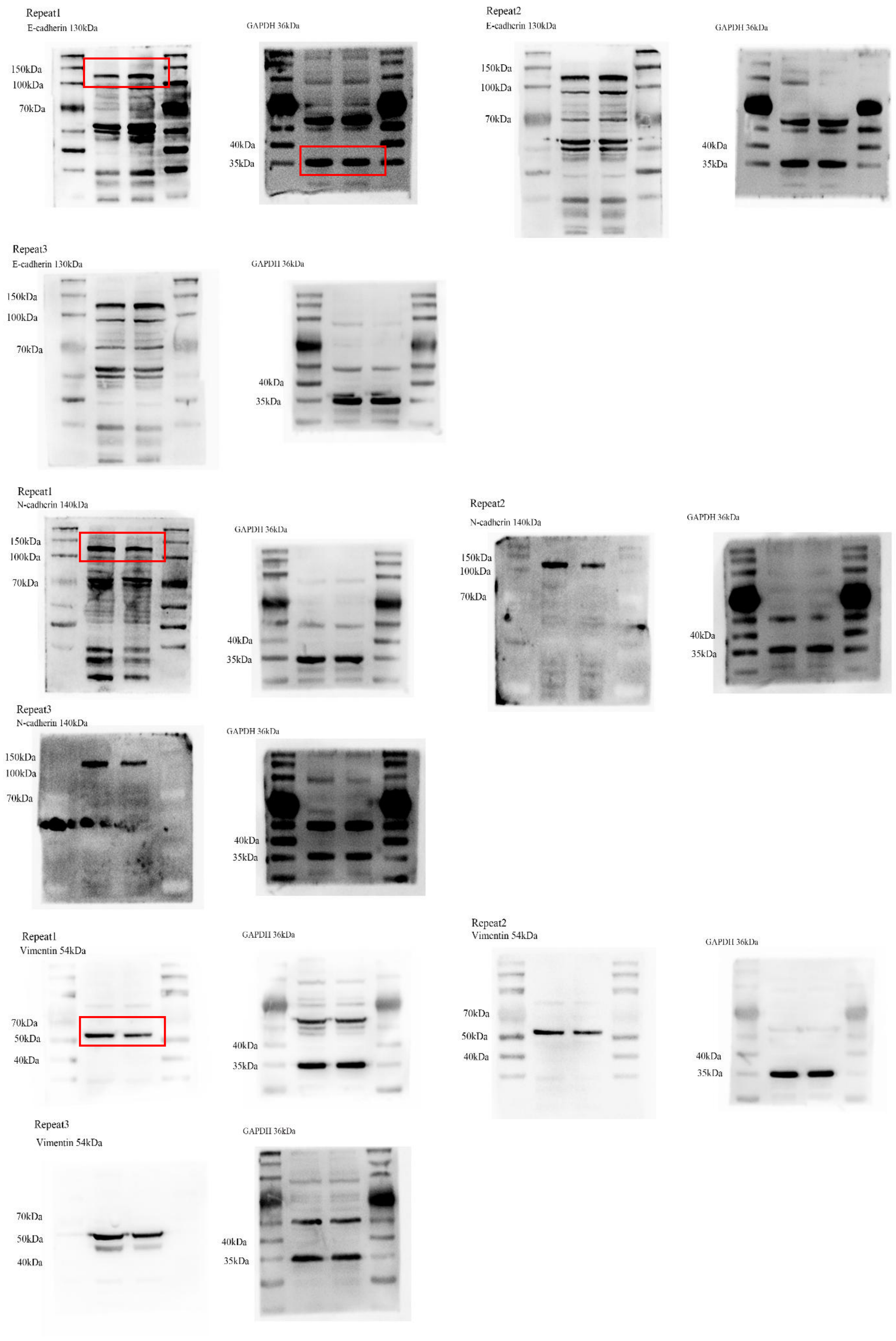

Original western blotting images for Figure 9C (E-cadherin,N-cadherin,Vimentin,GADPH)-SW620

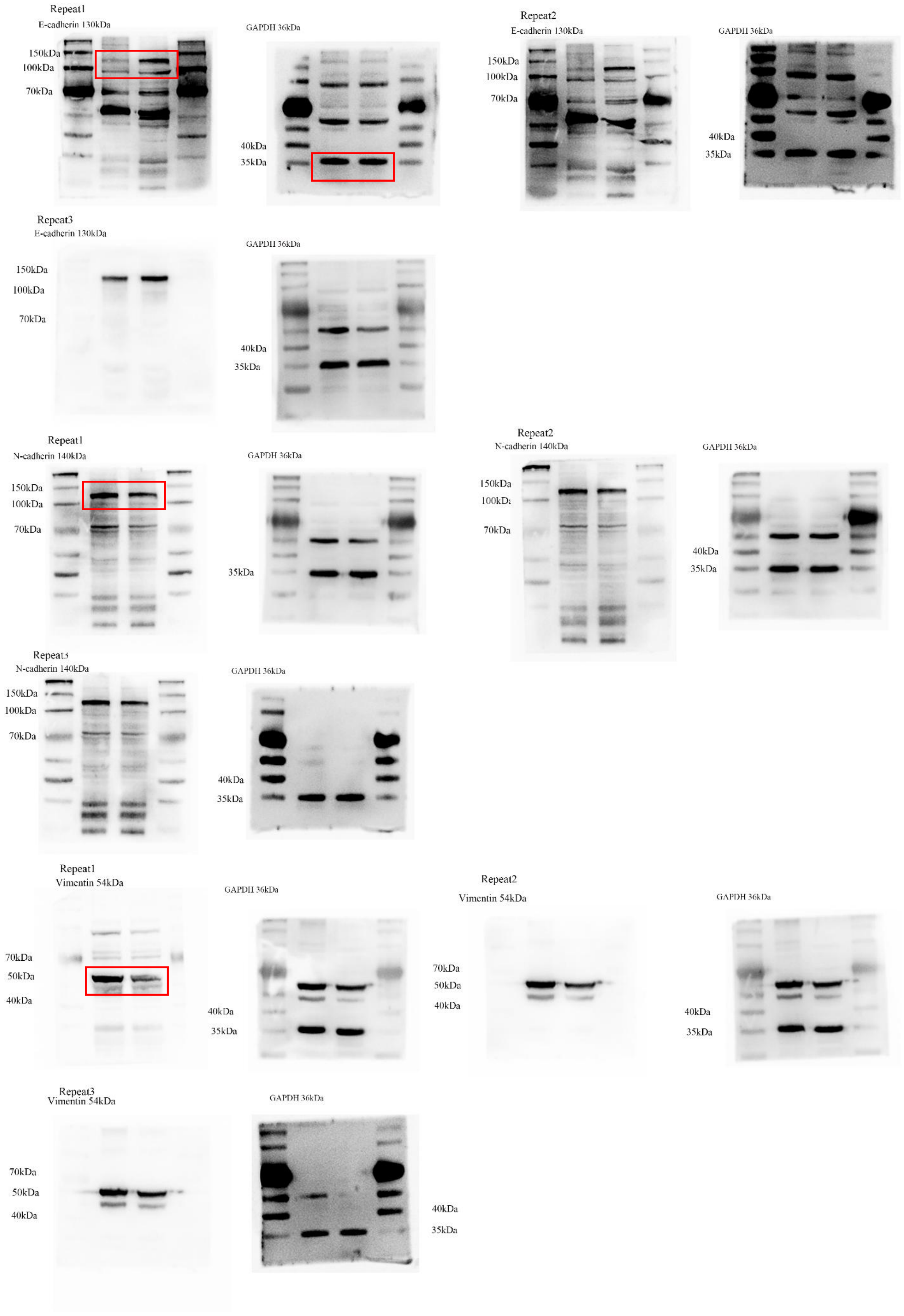

Original western blotting images for Figure 10A (p-Smad2,p-Smad3,TGF-βR1,GADPH)-HCT15

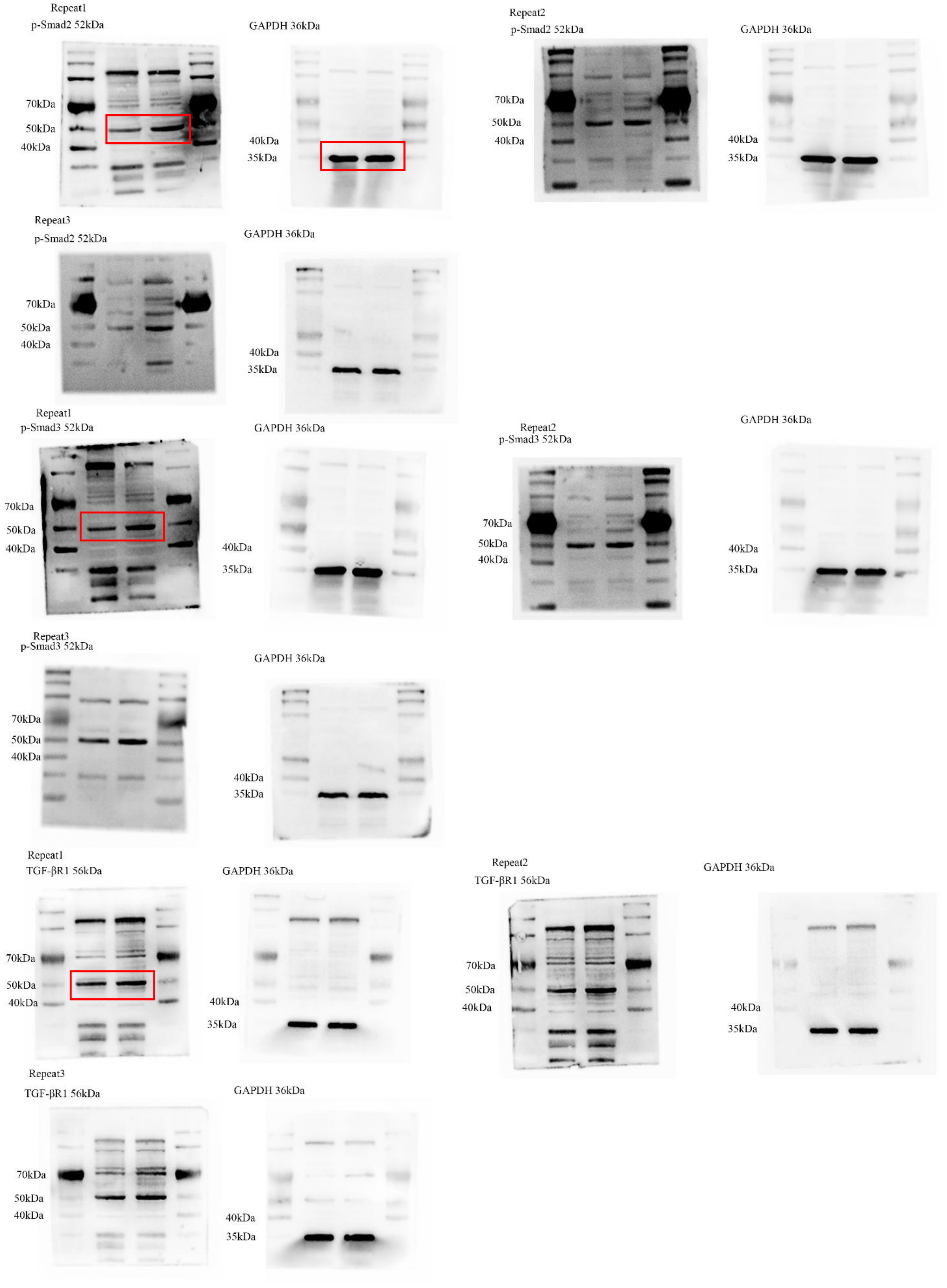

Original western blotting images for Figure 10B (p-Smad2,p-Smad3,TGF-βR1,GADPH)- SW480

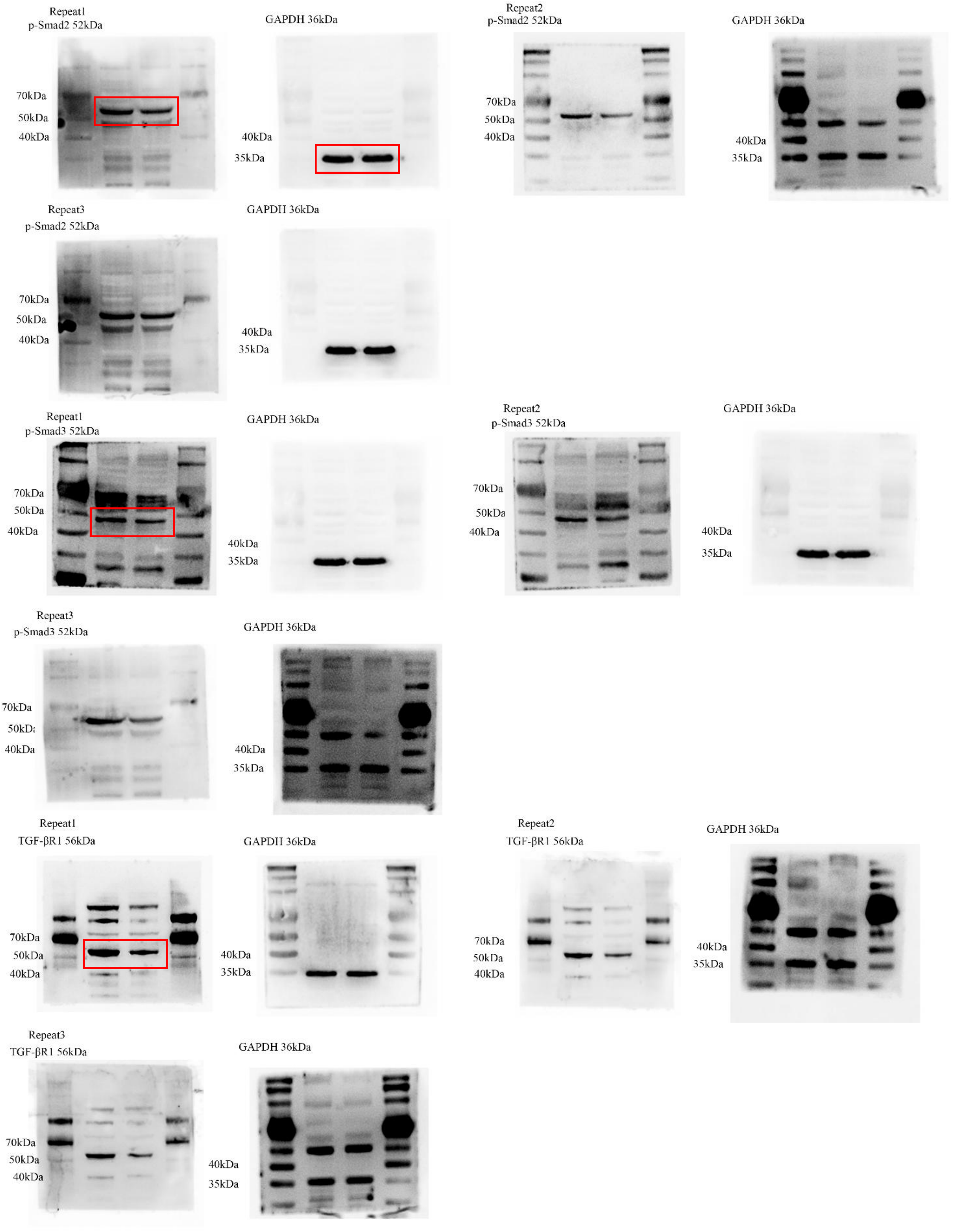

Original western blotting images for Figure 10C (p-Smad2,p-Smad3,TGF-βR1,GADPH)- SW620

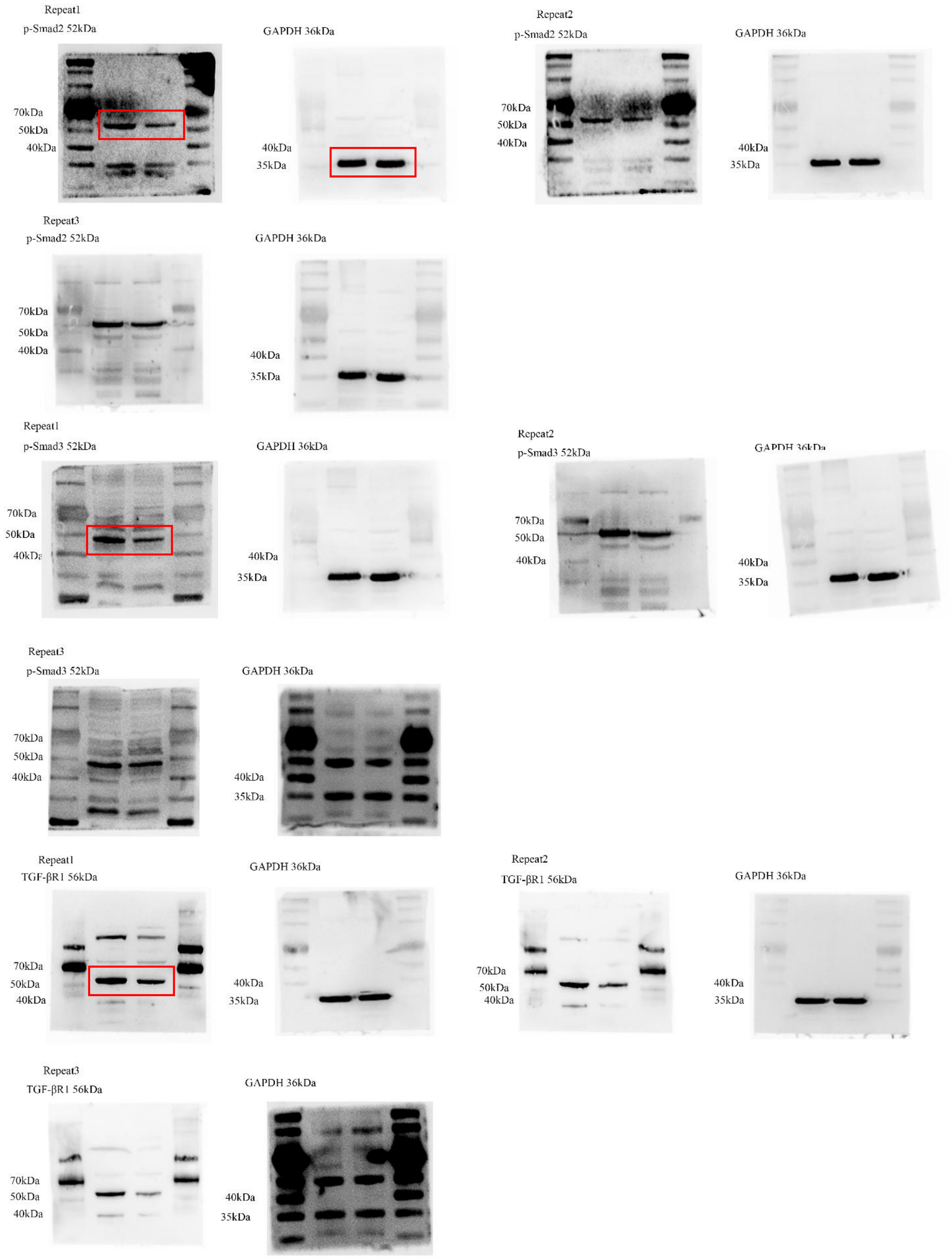

Supplement: Supplementary file 1 — Supplementary Material 1 [file 12885_2024_11925_MOESM1_ESM.pdf]
